# Predicting Cardiovascular Stent Complications Using Self‐Reporting Biosensors for Noninvasive Detection of Disease

**DOI:** 10.1002/advs.202105285

**Published:** 2022-03-24

**Authors:** Daniel Hoare, Andreas Tsiamis, Jamie R. K. Marland, Jakub Czyzewski, Mahmut T. Kirimi, Michael Holsgrove, Ewan Russell, Steven L. Neale, Nosrat Mirzai, Srinjoy Mitra, John R. Mercer

**Affiliations:** ^1^ Institute of Cardiovascular and Medical Sciences/British Heart Foundation University of Glasgow Glasgow UK; ^2^ School of Engineering Institute for Integrated Micro and Nano Systems University of Edinburgh Edinburgh UK; ^3^ BioElectronics Unit College of Medical Veterinary and Life Sciences University of Glasgow Glasgow UK; ^4^ Centre for Medical and Industrial Ultrasonics James Watt School of Engineering University of Glasgow Glasgow UK

**Keywords:** blood clot, cardiovascular disease, restenosis, stent, wireless impedance sensor

## Abstract

Self‐reporting implantable medical devices are the future of cardiovascular healthcare. Cardiovascular complications such as blocked arteries that lead to the majority of heart attacks and strokes are frequently treated with inert metal stents that reopen affected vessels. Stents frequently re‐block after deployment due to a wound response called in‐stent restenosis (ISR). Herein, an implantable miniaturized sensor and telemetry system are developed that can detect this process, discern the different cell types associated with ISR, distinguish sub plaque components as demonstrated with ex vivo samples, and differentiate blood from blood clot, all on a silicon substrate making it suitable for integration onto a vascular stent. This work shows that microfabricated sensors can provide clinically relevant information in settings closer to physiological conditions than previous work with cultured cells.

## Graphical abstract

An implantable miniaturized sensor and telemetry system is developed that can detect and discern different cell types associated with in stent restenosis, distinguish sub plaque components, and differentiate blood from blood clot. This work shows that microfabricated sensors can provide clinically relevant information in settings closer to physiological conditions than previous work with cultured cells.

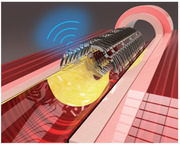

## 1 Introduction

### 1.1 Cardiovascular Disease Background

Cardiovascular disease (CVD) is the largest cause of human mortality in the world, with 17.5 million deaths per annum according to the World Health Organisation (WHO) and a cost of €210 billion per annum according to the European Heart Network.^[^ ^1^ ^]^ Two thirds of these deaths are caused by the build‐up of fatty material inside arteries termed atherosclerosis. The disease is driven by multiple risk factors that include high blood pressure, smoking, poor diet, genetics, and aging.^[^ ^2^ ^]^ Percutaneous coronary intervention (PCI)^[^ ^3^ ^]^ is the standard interventional treatment in which a metal stent is deployed via a balloon catheter to reopen the affected vessel segment, with over 3 million coronary devices deployed in the USA, Europe, and China per annum.^[^ ^4^ ^]^ Despite advances in technology a hyperplastic wound response termed in‐stent restenosis (ISR) often re‐blocks the stent in 5–10% of cases. Within 18 months these vessels can become reoccluded as cells slowly grow across the device struts. This leads to fatal reductions in blood flow and promotes clot formation that remains a significant global clinical challenge. Research has shown that ISR is driven by mechanical denudation of the single layer of endothelial cells (ECs) that line the vessel wall and the excessive proliferation of vascular smooth muscle cell (VSMCs) that form as a wound response.^[^ ^5^ ^]^ Exposing the medial VSMC layer of the artery to the bloodstream then promotes thrombosis and emboli that can enter the cerebral cardiac circulation to drive heart attacks or strokes. VSMC proliferation is accelerated by an inflammatory cascade that exacerbates vessel healing and leading to the recruitment of monocyte/macrophages.^[^ ^6^, ^7^ ^]^ These stented vessels can then suffer from neoatherosclerosis, which is the secondary formation of a lipid rich plaque with or without the calcification forming within a neointimal layer within the initial lesion.

To date many advances have been made in stent technology to overcome these complications. Initially bare metal stents (BMS) were replaced with drug eluting stents (DES) with a second generation of DES replacing them. This improved complication rates by reducing major adverse cardiac events (MACE) and the need for secondary intervention to treat ISR.^[^ ^8^ ^]^ DES have significantly lowered patient readmittance rates for target lesion revascularization (2.91% versus 12.32%).^[^ ^9^ ^]^ Yet with so many PCI procedures being performed globally,^[^ ^4^ ^]^ an estimated 100 000 patients still suffering life changing complications. Improvements in stent design such as reduction in stent strut thickness and drug elution coatings have also driven some improvements.^[^ ^10^, ^11^ ^]^ Technologies such as bioactive polymeric drug release systems that control coating thickness, surface roughness, drug load, and drug elution kinetics are also making significant improvements.^[^ ^12^ ^]^ However, there is no self‐reporting “early warning system” for stents that block and there are no intravascular stents able to detect the very earliest changes associated with vessel occlusion and the signs of thrombosis formation. The development of devices that can detect and report the dominant cell types involved and provide detection of early changes in real time from within the vessel is now becoming an increasing necessity.^[^ ^13^, ^14^, ^15^, ^16^ ^]^

### 1.2 Implantable Medical Device Concept

**Figure 1:**
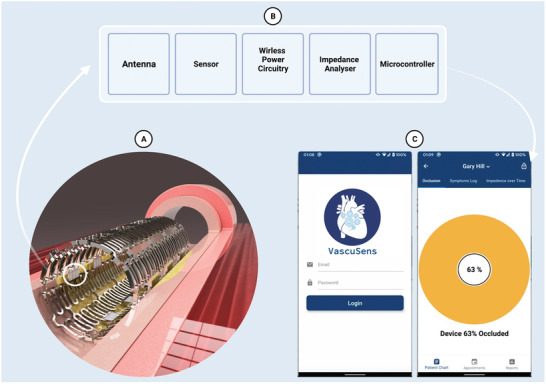
Conceptualization of a smart stent with multiple impedance sensors. A) The smart stent in situ of an artery will hold back a plaque that was originally blocking the artery, to make it a smart stent it will have multiple sensors incorporated throughout it to detect monolayer growth. This could also be expanded further to detect cross‐sectional reduction, pressure, and/or flow. B) Each “sensing” unit is an integrated chip consisting of a sensor, conditioning circuitry for control of a transceiver and impedance analyzer, an antenna, and a power unit which could be energized through wireless power to reduce the need for a battery on stent. C) This data can be relayed to through the cloud through secure connections allowing a doctor to securely log into a patient data to remotely assess the patient and provide treatment updates if necessary. The screenshots shown are of our Beta App, for example, patient name and data.

A self‐reporting device that can detect the blockage and its composition would be transformative for cardiovascular healthcare. A self‐reporting stent (SRS) would be predicted to lower the number of major adverse clinical events (MACE). A SRS would detect the earliest signs of the restenotic lesion and detect if this is a progressive VSMC rich lesion or a resolving EC rich one that would protect against thrombosis.^[^ ^17^ ^]^ Gathering clinically actionable data while the patient is outside of the hospital environment offers the opportunity of decentralised healthcare system.^[^ ^18^, ^19^ ^]^ This allows for a personalised treatment for each patient improving prognosis and supports the future of a stratified approach to cardiovascular medicine. In **Figure** **1**, we present our vision of how such a device could be achieved through electrical impedance spectroscopy (EIS) using sensors across a stent that report remotely to a physician through an iOS/Android phone application.

Electrical impedance spectroscopy (EIS) can be used to detect the varying electrical properties of human tissues. It has previoulsy been used for detecting and visualizing tissue structure abnormalities in the brain and lung^[^ ^20^ ^]^ and for detecting malignancies in vivo as well as in cell monolayers. A benchtop variant, electric cell‐substrate impedance sensing (ECIS) was previously developed by Applied Biosystems.^[^ ^21^ ^]^ EIS relies on an alternating current (AC) placed across a set of electrodes and using a range of frequencies at set intervals the impedance is measured. Impedance is the AC equivalent of resistance in a direct current (DC) circuit but in addition to resistance, impedance also describes the reactance across frequencies. In the context of detecting cell monolayers low frequency AC has been shown to flow through paracellular pathways, in between and around the gaps between the cells which act as insulators. In contrast at high frequencies the current flows transcellular, through the cell. This pathway can alter the impedance based on the cell type, intracellular composition, and structure that is present in and around the cell.

Previously we used relatively large 2 mm^2^ glass sensors with gold interdigitated electrodes (IDEs). Using the EIS method we were able detect the formation of a monolayer. Importantly, we showed the same electrodes could be used to induce a therapeutic intervention by inducing a controlled form of cell death termed apoptosis.^[^ ^22^ ^]^ These electrodes were oversized and made of glass making them unsuitable for further integration into a stent device. Stents are usually compressed onto a balloon catheter for delivery that needs to expand radially during inflation of a balloon, which is then withdrawn once the stent is in situ.

In this paper, we provide new evidence of a more realistic physiologically relevant model of cell detection and how this could be integrated into stents. Firstly, we moved the sensor from fracture prone glass substrates to a silicon substrate with 2 500 µm^2^ IDEs made of platinum. Next, we investigated the sensors suitability for future clinical use evaluating its ability to distinguish ECs and primary aortic smooth muscle cells (SMCs) in vitro, the two predominant cell types involved in ISR. This is important as it allows the detection of the predominate cell type for future therapeutic intervention of the VSMC rich lesions that drive ISR. Furthermore, we assessed the sensors ability to function in the presence of blood and explanted human carotid plaque specimens ex vivo. Finally, to move toward a realistic deployable device we custom designed our own nitinol stent with a unique platform that allows the sensor to remain in situ during radial expansion without deforming. This sensor element was then attached to our custom miniaturized implantable impedance telemetry package for wireless cell reporting to a handheld Android/iOS App.

## 2 Results

### 2.1 Investigation into Cell Discrimination

**Figure 2:**
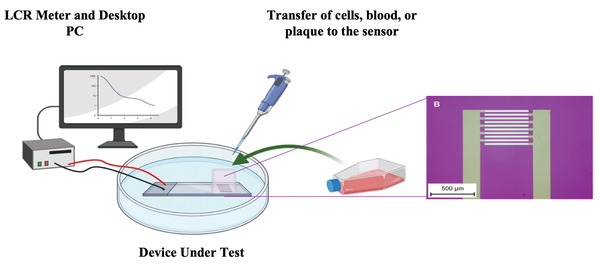
Experimental set up for device under testing. Cells are transferred in the required density from a tissue culture flask and pipetted into the sterile chamber in which the functional area of sensors is. A LCR meter is attached to the sensor and the resulting impedance over a frequency spectrum determined and analyzed using a personal computer (PC). Enlarged optical image of tested silicon interdigitated electrodes (IDE) is shown. (Created with BioRender.com)

**Figure 3:**
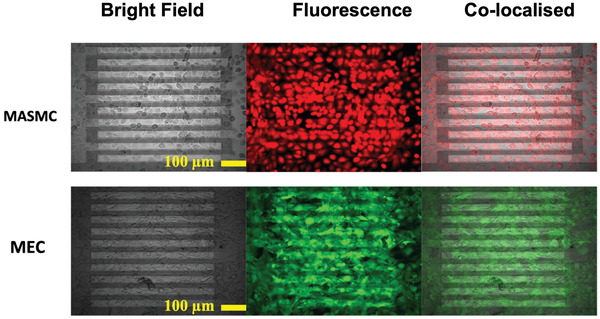
Brightfield and fluorescent images of cell monolayer on silicon sensors. Mouse aorta smooth muscle cells (MASMCs) are shown in the top panels with red Calcian AM dyes. Mouse endothelial cells (MECs) with calcian AM are shown in green on the bottom panels.

**Figure 4:**
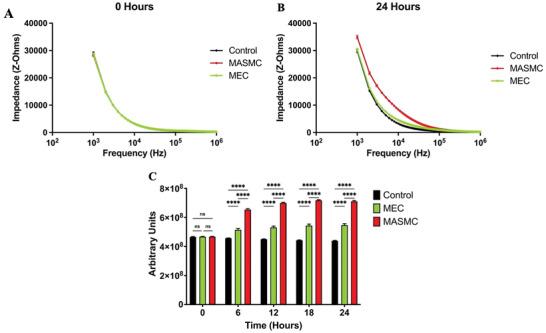
Impedance values of controls vs. mouse aortic smooth muscle cell (MASMC) vs. mouse endothelial cell (MEC) at select time points. A frequency sweep between 1 KHz–1 MHz is shown at 6‐h intervals for control is shown in black, 50 000 MASMCs identified by the red line and 50 000 MECs represented by the green line, panels A and B. In Panel C, the area under the curve (AUC) of these sweeps at each time point is presented with comparisons between each group. MASMCs = Mouse aorta smooth muscle cells; MECs = Mouse endothelial cells. Mean ± SE, *n* = 6, ns = nonsignificant, ^****^ *p* < 0.001. Two‐Way ANOVA with Tukey.

Silicon sensors were seeded with 50 000 primary mouse aortic smooth muscle cells (MASMC) or 50 000 mouse endothelial cells (MECs) or a media only control, see experimental set up **Figure** **2**. There was no observable difference in monolayers between the control and sensors seeded with the different cell types. The monolayers for MASMC and MEC at 24 h are shown in **Figure** **3** under brightfield and fluorescent dyes (Calcein, ThermoFisher Scientific). Impedance measurements were then made from 0–24 h at 6 h intervals across a range of frequencies. Initially at 0 h, there was no significant difference in measured impedance (1 kHz–1 MHz) between the control and the sensors seeded with the different cell types (**Figure** **4**A). However, after 24 h the impedance sweeps at 1 kHz–1 MHz showed clear differences with MASMC stained red, significantly exceeding the values of MEC, green, and controls, black (Figure 4B). Importantly cell viability and cell counts confirmed this was not due to changes in rates of cell division or cell death. Performing area under the curve (AUC) analyses for the impedances across the 1 KHz–1 MHz sweeps confirmed these significant differences between the control and MASMC or MEC and also between the MASMC and MEC groups between 6 and 24 h, Figure 4C, *p* ≤ 0.0001. This supports our finding that different cell types seeded in identical conditions have different measured impedances. We believe that this would be a methodology for discerning the very earliest signs of the of the initial restenotic growth response of a monolayer on a stent or graft and for the detection of re‐endothelialization through MEC.

### 2.2 Assessment of Blood and Clot

**Figure 5:**
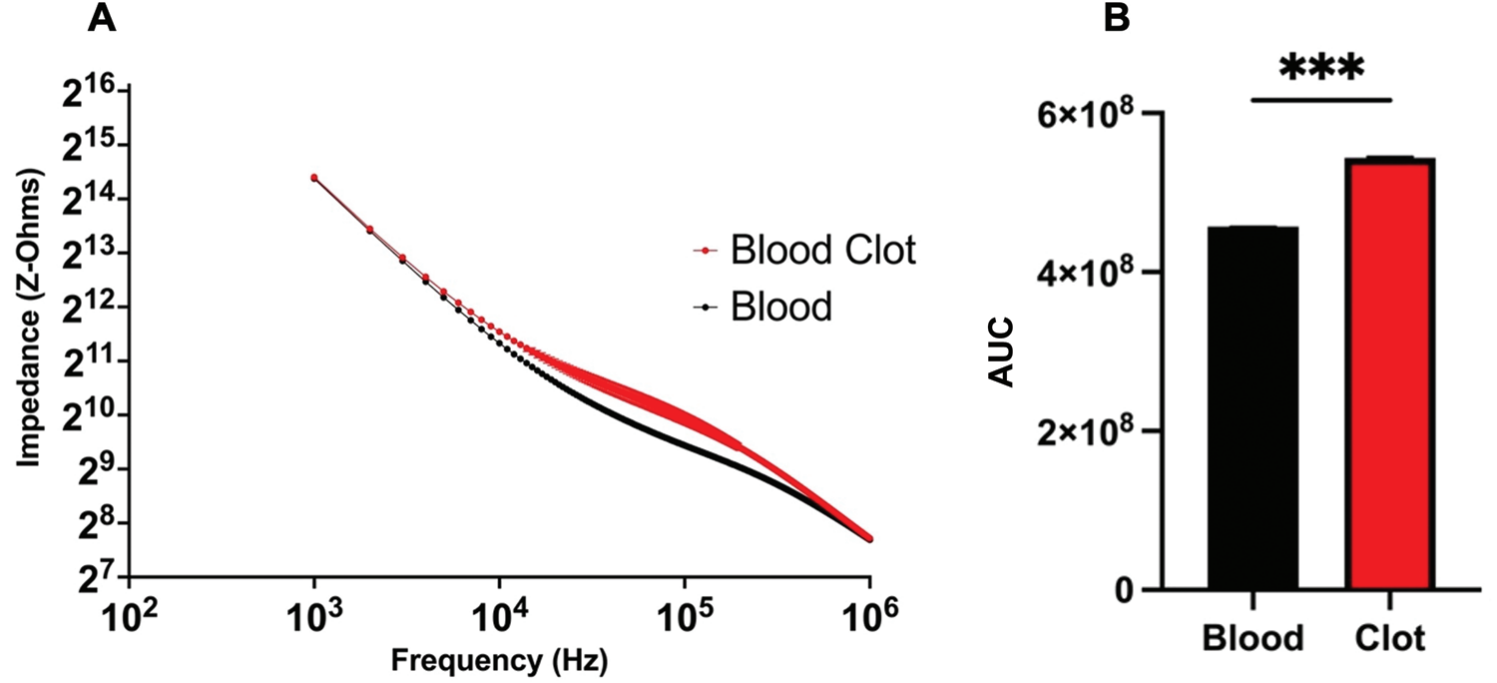
Impedance analysis of heparinized blood versus clotted blood. Panel A shows the impedance of the samples across a frequency spectrum of 1 KHz to 1 MHz. Panel B shows the area under the curve (AUC) of the samples, *N* = 3, ±SE, Welch's T‐test. ^***^ *p* < 0.001, *t* = 64.07, df = 2.032.

The combination of a sensor to a stent means it will be in contact with blood. As such the sensor must be able to discern not only the growth of EC and SMC growth but also other constituents of the intravascular in vivo environment. As such measurements of heparinized blood, that is prevented from clotting, with clotted blood were made in vitro. In **Figure** **5**A the impedance of these is shown across a frequency spectrum with comparison of AUC in Figure 5B. After coagulation of the blood the impedance was greater than that of the heparinized blood over the range 10 to 500 KHz. This is confirmed by comparing AUC analysis within the samples where a significant difference can be observed, *p* = 0.001. The early and remote detection of blood clot is important in a number of clinical pathologies and the ability to then mediate blood clot lysis therapeutically could be equally useful and is discussed later. Combining the ability to detect either a SMC rich lesion or re‐endothelization with blood clot detection is a potentially innovative use of EIS especially within the complex multicellular environment of vascular lesions.

### 2.3 EIS of Human Carotid Endarterectomy Specimens

**Figure 6:**
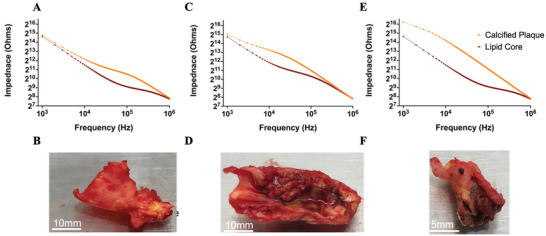
Impedance analysis of lipid core versus calcified cap with corresponding plaque. Panels A and B show mild atherosclerosis, panels C and D show moderate atherosclerosis and panels E and F show severe atherosclerosis. *N* = 1.

It is apparent that the use of purely in vitro monolayers to replicate the in vivo conditions is limited as it does not fully reflect the multicellular composition of vascular lesion in vivo. To address these issues we tested our device using human carotid endarterectomy specimens ‐ complex atherosclerotic lesions with varying disease, see methods for ethics approval. To validate our approach human plaques were visually inspected, and ranked according to American Heart Association (AHA) criteria for disease severity (**Figure** **6**). The plaques classification was based on the degree of vascular occlusion caused by the impinging plaque into the lumen as; mild stenosis (Type IV) moderate (Type VII), and severe (Type VIII) lesions.^[^ ^23^, ^24^ ^]^ Each lesion was then microdissected and divided into calcified rich portions and separated from regions containing the fatty lipid rich core. Electrical impedance measurements were then undertaken with luminal portions of each plaque in contact with the sensor elements (Figure 6A–F). The lipid core showed similar impedance across the spectrum in magnitude and character, but for moderate disease the impedance was slightly greater than the others from 10 to 100 KHz (Figure 6C). The calcified cap impedance was increased compared to the lipid core, with the difference between the magnitudes being more apparent at the lower frequencies as the disease became more severe (Figure 6A,C,F). This difference is thought to be due to the differences in the volume of calcification, although a slight frequency response regarding volume increase has previously been shown for low frequency AC signals.^[^ ^25^ ^]^ The increase in impedance between the lipid core and the calcified cap is due to the permittivity of the calcium within the plaque being greater than that of the lipids. As such the detection of neoatherosclerosis by sensors within a stent could be possible.

### 2.4 Stent Integration and Testing

**Figure 7:**
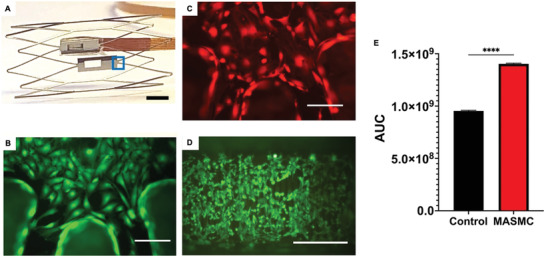
Integration of silicon biosensor on custom Nitinol stent for cell detection. a) Custom dual platform of an electropolished Nitinol stent (scale 2 mm) with a sensor facing to the lumen of the artery. Fluorescent calcein dyed cell across stent struts and sensor surface. b) Mouse endothelial and c) MASMC, mouse aorta smooth muscle cell ×20 (150 µm). d) Monolayer growth after 24 h on a sealed and assembled stent with sensor, sensor area shown by blue box. e) 24‐h comparison of control (media only) versus 500 000 MASMC via AUC of impedance over 1 KHz to 1 MHz. Welch's T‐test. *N* = 3 ±SE, *p* ≤ 0.0001, *t* value = 52.03, df = 4.

Having shown the ability to discriminate cell types in vitro and sub plaque components ex vivo we then integrated our sensors into a custom nitinol stent that integrated a non‐deforming sensor platform (**Figure** **7**A). Nitinol is a corrosion resistant and memory alloy that is ideal for the placement of the sensor on the stent and that is capable of being radially expanded without deformation as per PCI in patients. The non‐deformable platform measures 2 × 5 mm with a “sensing” window of 2.1 × 1 mm. The stent pattern and diameter were deliberately generic (10 mm with a length of 20 mm) and custom designed to accommodate our current sensor. The sensor islands or window allows for the orientation of the IDEs to be facing toward the luminal side of the artery, but could equally be reversed for abluminal or wall detection (see section 3.5), while the excess bulk of the sensor is clear of the lumen and embedded within the stent structure. Bonding of the sensor to this type of stent means the sensor island is less prone to damage and as such failure of the sensor to the stent is less likely. Equally, we endeavor in creating such a device that the materials used should be biocompatible and not induce cytotoxic reactions.

Importantly, previous computational modeling has shown the cross‐sectional profile of our stent to contribute no greater resistance to blood flow than current marketed stents with a stent strut profile of <100 µm but yet the design is scalable to accommodate a number of endovascular stent sizes. To validate the stents biocompatibility, we cultured both VSMC and ECs upon the stent surface confirmed their viability (Figure 7B,C). A miniaturized version of the senor was then fully integrated to the stent and a stained monolayer of MASMCs grown across the sensor after 24 h is shown (Figure 7D.) Monolayer formation across the stent and in situ sensor was assessed through impedance measurements across a 1 KHz–1 MHz range. Using AUC analyses the impedance had increased significantly compared to the control of media only after 24 h, *p* ≤ 0.0001 (Figure 7E).

### 2.5 Implantable Impedance Telemetry Development

**Figure 8:**
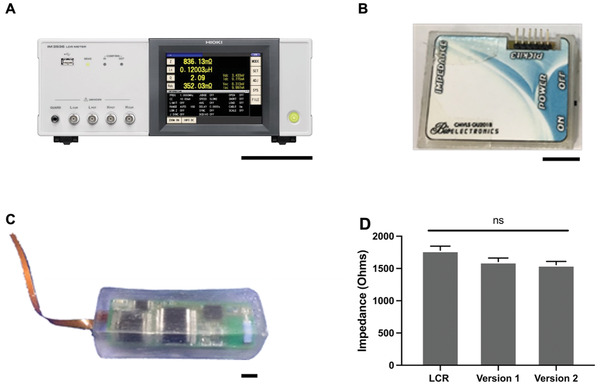
Comparison of impedance analyses. Panel A shows a bench top LCR meter used in the experiments, scale bar 10 cm. Panel B shows version 2 of the in house wireless impedance telemetry based of a AD5933 impedance chip, scale bar 10 mm. Panels C shows version 3 of the wireless implantable telemetry with a miniaturized triple layer printed circuit board (PCB) in biocompatible acrylic. Panel D shows the comparison of the two generations of miniaturized wireless impedance analyzers to LCR, *N* = 3, ±SE, ns = nonsignificant, one‐way ANOVA, *f* value = 4.417, df = 8.

During the testing we used a bench top LCR meter, see **Figure** **8**A, however in order to be implantable this needed to reduce in size. To report the impedance from the sensor‐stent we developed a miniaturized telemetry device based on an AD5933 (Analogue Device). Version 1, Figure 8B the printed circuit board (PCB) was reduced to 5 cm by 3.5 cm. In version 2 a triple layer PCB of 3.5 by 1 cm was made and encapsulated in biocompatible acrylic (Blanson Ltd, UK), Figure 8C. Both versions 1 and 2 were tested against the gold standard of a LCR meter (Hioki LCR Model im3536) under a test load of 50000 MASMC. Comparing the devices at 20 KHz, arbitrary point, the devices were not significantly different (Figure 8D). Integrating the sensor, stent and telemetry was a significant undertaking yet we were able to significantly miniaturization the performance of a wireless impedance analyzer while maintaining high quality impedance measurements with version‐2, 91.8% ±0.003, and version‐3, 89.5% ±0.007, of the LCR meter. These telemetry devices are capable of relaying data through a base station which then uploads the data to a secure cloud server which can be accessed by an App. In the future, it is aimed that a wearable device will be able to access the implanted stent to then transfer the data to the cloud rather than use a base station. The App shown in Figure 1C and is capable of accessing this data and automatically graphing the data, however quantification of an occlusive blockage is still an aspirational target that needs further investigations to achieve this.

## 3 Discussion

### 3.1 Appropriateness of EIS for Cell Discernment

Although electric impedance spectroscopy is well established and used to investigate cell and cell monolayer interactions it has yet to be appraised for implantable medical devices such as proposed in our work. Classification of different cell types has also previously been achieved with a focus on comparing healthy and cancerous cells.^[^ ^26^ ^]^ In vitro research has shown that cell types produce unique impedance signatures and thus can be determined from their signatures in a label free way.^[^ ^27^, ^28^ ^]^ For example detection of undifferentiated stem cells and their transition to bone like osteogenic or fat like adipocytes cells.^[^ ^29^ ^]^ Using EIS as for mislabeled or unlabeled cell lines mammalian cell lines has also been achieved.^[^ ^30^ ^]^ Using out of sample prediction, a form of machine learning, it was possible to identify and group the cells, which the authors reporting an accuracy reaching values of 97% and 99%, respectively. Holland et al. also investigated EIS for ISR, they used large working electrodes with an oversized counter electrode to evaluate SMCs and ECs in vitro that however these were not suitable for in vivo applications nor had an implant or data telemetry device been developed.^[^ ^31^ ^]^ Like our work they identified that the impedance of the two cell types differed, hypothesizing that like other researchers the formation of cells tight junctions, cells morphology, and strength of adhesion played key roles in the detection.^[^ ^21^, ^32^ ^]^ In our work the changes in impedance are hypothesized to be due to the morphological changes of the cells and how those cells spread across the sensor surface. However, what has not previously been achieved to date is the integration of a miniaturized impedance sensing element with an implantable device capable of wirelessly reporting clinically important data. Moreover, the proof of concept presented here is amenable to a number of other pathologies in which cell type and clot detection are important.

In addition to restenosis, clot detection is a particularly important condition to detect and reflects both chronic and acute pathologies. Firstly, during a PCI procedure, clot can be triggered by equipment used to deploy a stent, poor anticoagulant therapy, or the inflation of a balloon or stent in the vessel.^[^ ^33^ ^]^ Secondly, post‐stent implant the rupturing of neoatherosclerosis or inducing ISR can lead to the formation of a blood clot. We found using heparinized blood better represents the conditions in which a stent nominally operates as fresh blood spontaneously clots in air. We show that clotted blood has an increase in impedance compared to whole blood when measured using our miniature IDEs. Ramaswamy et al. using gold electrodes on a glass and polydimethylsiloxane (PDMS) device reported the impedance increase as a blood clot formed.^[^ ^34^ ^]^ This effect was also shown by researchers assessing hematocrit, defined as the blood cell volume. Hematocrit levels change in response to temperature, effecting the clotting time but impedance of a clot was found to be always be greater than that of a blood alone.^[^ ^35^ ^]^ Similarly to the EIS research these devices are not suitable for implantation due to a lack of deployment device, the materials use as glass can fracture, and the size of the device. Using our sensor combined with a stent or similar device we would be able to detect early thrombus formation during a stent deployment and as well as postsurgery. In detecting the early changes of clot formation intervention could be taken quickly such as use of blood thinning medications before complications ensue.

In addition to cell and clot detection we have shown that it is possible to discern different cellular components between human plaque samples. Compared to normal vessels, lipid rich disease prone vessels have been shown to have an increased impedance, this was confirmed with ultrasound imaging and histology of the vessel.^[^ ^35^ ^]^ In a further balloon mounted impedance sensor in vivo data showed an increased impedance dependent on the lipid cores constituents and fibrous cap with stable blood impedance measurements.^[^ ^36^ ^]^ In a model by Packard et al. (2016) an in vivo study using rabbits vessel and a stretchable balloon with mounted sensors was presented. They showed that severe plaques had greater impedance over moderate plaques and mild plaques to be the lowest impedance of all.^[^ ^37^ ^]^ This was similar to the results we obtained in the calcified portion of the dissections where by the impedance was greater in more severe lesions. Our work has the distinct advantage of being an implantable and permanent reporting device that will monitor a patient at regular intervals over the lifetime of the device. Therefore, this implantable device allows for ambulatory care while the patient is out of hospital rather than a snapshot at time of implant. Further work is needed with ex vivo and in vivo measurements to confirm these benefits for our work.

### 3.2 Value of Sensors Integrated into Stents, Grafts and other implantables

Integrating a platform to a stent with a sensor and electronics has been shown to be a viable method for stent sensor integration but one which is equally amenable to other implantables such as synthetic grafts or stent graft hybrids.^[^ ^38^, ^39^, ^40^, ^41^ ^]^ For example the work of Park et al. with pressure sensors shows stents having platforms at either end of the stent that exceed the length of the stent may be a solution.^[^ ^13^ ^]^ Takahata et al. turned the stent into the antenna adding sensor platform at the end of the stent once more, by using the stent as antenna reduced the amount of electronic components marking.^[^ ^15^, ^42^, ^43^ ^]^ Our device differs significantly, firstly our sensor detects monolayer changes which are the earliest sign of ISR therefore allowing for treatment before a cellular blockage or clot forms. Secondly the device can discern the difference between reendothelization which a physician would wish to promote or VSMC hyperplasia which a physician would wish to suppress. Both of these pathologies are common in implantable stents and vascular grafts where the implant is in contact with the native vessel. Having the ability to discern the cell types may revolutionize the way they treat the patient in the short and long term by medication changes and reduced hospital visits. The previous researchers pressure changes are specific to later stage disease where a narrowing of the artery has already occurred to cause this pressure and flow changes. As such interventions are more limited at this later stage and could have already induced downstream ischemia. Further, unlike Park et al. for example, our stent is made of clinically approved nitinol and based off a pre‐existing clinically approved design, albeit a simple and oversized design for the coronary artery. This is an improvement due to the better conformity than a 3D printed stent and the nitinol does not deform keeping the sensor in place. Furthermore, our device has a low power telemetry device that reports to an app through a cloud based storage allowing for secure and rapid result reporting. Using a cloud based solution will allow for future machine learning tools to be deployed to automatically discern cell types and report to the app. Moreover, our experiments have used explanted plaques a step no other group, to our knowledge, has used to validate a smart stent thus making it the first to use physiologically and biologically relevant tissues to evaluate a smart stent sensor. While the use of EIS is a common laboratory practice the mounting of such a sensor, with miniaturized on telemetry onto a custom medical grade stent is not common and has distinct advantages over other research in this field.

When coupled with our prior therapeutic work the conversion and miniaturization to silicon sensors paves the way for a future self‐reporting stents or grafts capable of assessing internal conditions and treating ISR. Indeed, the current stent dimensions are larger than that of the coronary arteries as original conceived, but the device is close to lending itself to a number of other peripheral vasculatures applications such as arteriovenous graft occlusion, carotid atherosclerosis, aortic aneurysm detection, catheter blockages, oesophageal stricture, tracheal blockages, and many others where the detection of cellular growth and clot detection is clinically useful.

### 3.3 Clinical Relevance

The formation of a blood clot or significant accumulation of cellular material are costly complications if they occur after implantation of a stent. To date, drug coatings on the stent and dual antiplatelet therapy will remain the gold standard of treatment to reduce these eventualities. However, the ability to detect the formation of these adverse events at the cellular level rather than waiting for occlusive blockage is a distinct advantage in our proposed design. A diagnostic and therapeutic system offers the opportunity of a new class of “closed loop” of implantables devices amenable to detect and treat a number of cardiovascular pathologies where cell detection during wound healing is important. The proposed surveillance offered by an early warning system like this means timely intervention can be taken, perhaps even outside the hospital environment in the so‐called “decentralized healthcare system.” Unlike other researchers work that rely purely on the flow and pressure disturbances caused by large occlusions, we deliver a system with the potential to flag the very earliest signs of impending complications weeks or months before they form and become a clinical problem. Our device not only warns, it reassures which in a disease that is clinically silent offers great implications for the future of such devices.

### 3.4 Sensor and Stent Integration Relevance

In this paper we integrated a packaged silicon sensor on a nitinol stent. We orientated the sensor to be facing into the lumen and placed two nondeformable platforms in the center of the stent. The mechanical forces exerted on a stent during the crimping and expansion periods are high. However, the unique platform placement and the use of nitinol is an important factor for preserving the platform and sensor during radial expansion. The silicon we used is inherently fragile but strengthened by encapsulation to withstand the stretching and flexibility that a stent undergoes in vivo.

The orientation of the sensor is an important factor. If orientated to face the abluminal wall (the wall of the vessel) you could detect growths that were not clinically relevant as it would always be in contact with potentially pathological cells. With the sensor orientated to the luminal side, one is able to detect the formation of a blood clot in the lumen but also the migration of cells from and around the vessel wall into and onto the stent. As the stent is a large object it protrudes in the lumen, therefore cell build up is greater than the stents profile and thus important to detect. The VSMCs of the wall will migrate from the site of placement and attach to the stent and continuing proliferating to cause a restenotic blockage, while endothelial monolayers are predicted to silence VSMC response to injury. As such, orientating the sensors toward the lumen is preferred for detecting monolayers of different cell types. Additionally, if the stent undergoes re‐endothelization this would be detected as the cells migrate across the stent and sensor. If more than one sensor is used than this orientation could also allow for the detection of multiple layers of cellular material to be detected.

### 3.5 Future Considerations

With the complex nature of the human body one rightly founded concern would be how we control the episodic application of the small current and voltages applied by the sensor to the vascular wall, and how this might affect the intrinsic electrical system of the heart. The coronary arteries are on the surface of the heart and the stent and sensor would be within the artery wall which is made up of multiple layers with varying permittivity and conductivity. The sensor itself would be encapsulated and electrically isolated except for the sensor window and importantly in a nondeformable area of the stent for crimping and expansion. Never‐the‐less, to assess the safety of our design we recommend using an isolated heart “Langendorff” perfusion setup prior to any in vivo tests. In addition, the research would benefit from a comparative study to investigate the difference in human VSMCs and ECs and see if these trends identified here hold true across species. If successful, preclinical validation studies in vivo would be needed to progress the device to the next stage of development.

## 4 Conclusion

In this paper, we have optimized a way of discerning cell types in vitro by use of an IDE sensor on silicon. We have shown how changing the dimensions of the sensor from previously published work on glass to silicon significantly improves the sensors capabilities and allows for ease of future scalable manufacturing. Through the use of impedance magnitude, we have shown a way in which cell types can be independently discriminated from each other by three different evaluation methods. We have also shown that the sensor can be miniaturized and packaged to an implantable stent, thus providing potential future translational application to an IMD capable of detecting and differentiating cell types early in order to combat in stent restenosis and detect thrombosis. Future plans are now underway to complete in vivo validation of our design.

## 5 Experimental Section

### Fabrication of Interdigitated Electrodes

Silicon IDEs were fabricated on 100 mm n‐type <100> silicon wafers using standard microfabrication techniques. IDE measurements were width 25 µm and length 500 µm with 25 µm spacing for five pairs. A 500 nm thick bottom insulating layer of silicon dioxide was grown by wet thermal oxidation. The wafers were then treated with vapour HMDS adhesion promoter for 10 min and spin‐coated at 4000 rpm for 45 s with nLOF 2035 negative photoresist, followed by a soft bake at 110 °C for 60 s. A MA8 mask aligner (Suss Micro Tec) was used to expose the wafers for 15 s through a chrome photomask comprising the IDE patterns, followed by a post exposure bake at 110 °C for 60 s. The coated wafers were then submerged in AZ 726 developer for 2 min and rinsed with de‐ionized (DI) water, prior to a 5 min oxygen plasma clean. 10 nm of titanium adhesion layer and 50 nm of platinum were then deposited by thermal evaporation and the IDE, interconnect and contact pads were patterned by lift‐off using 1165 photoresist remover followed by rinses with IPA and DI water and a 30 min O_2_ plasma. A 500 nm thick silicon nitride (SiN) top insulating layer was then deposited using plasma enhanced chemical vapour deposition. To pattern the SiN, the wafers were first spin coated at 2000 rpm for 30 s with SPR 350 positive photoresist, followed by a soft bake at 90 °C for 90 s, lithographic exposure for 15 s, post exposure bake at 110 °C for 90 s, and photoresist development for 1 min using MF 26A developer. The SiN layer was then etched for 6 min on a CF_4_ and O_2_ plasma, using reactive ion etching. This process step produced openings that define the platinum IDE and contact pads, while the interconnect remains insulated. Remaining photoresist was then removed using 1165 photoresist remover, and the wafers rinsed with IPA and DI water. The wafers were finally diced to 11 × 25 mm chips.

### Cell Culture

Commercially available Perspex 8 well culture chambers (Falcon 354 118, Thermo Fisher Scientific, United Kingdom) were separated into individual chambers and bonded with an ultraviolet (UV) curable acrylic adhesive (Loctite AA 350 UV, Henkel Limited, Germany) onto the sensors. These were cured for ≈2 min on a UV stage with built in heater until the glue was solid. Prior to cell plating each sensor was handled in a sterile fume cupboard, treated with 650 µL of 70% ethanol and then rinsed with DI water three times before 650 µL of Dulbeccos Modified Eagles Medium (DMEM) HEPES buffer (Gibco, UK) supplemented with 10% foetal bovine serum, 1 mL per 100 mL penicillin/streptomycin and L‐glutamine was applied to them only for controls. Devices were incubated at 37 °C and buffered with 5% carbon dioxide at 95% relative humidity in the absence of any cells for control readings (Figure 6). Afterwards the devices had the media removed and were cleaned again with DI water three times. MASMCs from an adherent transgenic mouse model, as previously described,^[^ ^44^ ^]^ were seeded in a 650 µL suspension to each sensing devices culture chamber, in the desired seeding density. They were incubated in accordance with each experiments time points. This was repeated for immortalized MECs, as previously described.^[^ ^45^ ^]^

### Sensor Encapsulation and Integration with a Custom Stent

A custom stent was designed using SolidWorks (Dassault, France) and Fusion 360 (AutoDesk, USA). Prototypes were rapid prototyped on a ProJet 1200 SLA printer (3D Systems, USA). A custom Nitinol self‐expanding stent was laser cut with an electro chemical polishing by Meko (MeKo GMBH, Germany). Knowing that silicon sensors with platinum IDEs were capable of cell detection these were packaged into a compact sensor and encapsulated for added protection.^[^ ^46^ ^]^ A custom stent was created through computer aided design and rapid prototyped through in house 3D printing until a final design was realized. This was passed onto a commercial laser cutting company (Meko, Germany) who created a Nitinol stent with an electropolished surface finish measuring 10 mm internal diameter by 20 mm with a strut thickness of 160 µm the island that holds the sensor 5 × 2 mm 2.1×1 mm hole Figure 6A. The sensor was exposed onto the luminal surface of the stent using a biocompatible epoxy (UV15×6MED‐2, Masterbond, USA) and seeded with MASMCs and MECs.

### Human Tissue and Blood Collection

Patient consented human carotid endarterectomy tissue samples were provided under license from the NHSGGC Biorepository and Pathology Services (REC 10/S0704/60) after approval from the West of Scotland Research Ethics Service. Human carotid arteries endarterectomy samples were divided into three portions. One explanted for derivation of fresh plaque VSMC lines, a second was paraffin blocked for histology, and a final unfixed portion stored in −80C. These fresh/frozen samples were easily classified into hard calcified sections and soft noncalcified sections during the initially specimen processing and then directly applied luminal surface down to the sensors.

### Fluorescent Staining and Imaging

Olympus X71 with CoolLED fluorescent light source was used to image the device a QImage camera. Digital images were acquired using the QImage Pro software suite. Overlays were created using ImageJ open‐source software with no additional plugins. Cells were stained using Calcein AM (green) and Calcein red orange (red) (Invitrogen, Thermo Fisher Scientific, USA, C34851 and C3100MP). The media was removed and washed with 200 µL phosphate‐buffered saline (PBS) (Gibco, Thermo Fisher Scientific, USA, 14 190 144) to remove all traces of serum, the PBS was then removed. An amount of 2 µL of dye was dispersed with 198 µL of culture media to a final concentration of 10 × 10^−6^ m. MECs were stained green and MASMC stained red. Cells were incubated for 30 min, and dyes were removed. Cells were washed with PBS and the culture well refilled and imaged.

### Impedance Measurements

Impedance measurements were made using a commercially available Hioki IM3536 LCR meter (Hioki Corporation, Japan). Measurements were made at identical time points across controls and experimental devices at regular time points. LCR meter exports data as comma separated values and was handled using Excel Microsoft Office (Microsoft, USA).

### Statistical Analysis

Data were analyzed and presented using GraphPad Prism version 9 (GraphPad Software, USA). Throughout data were presented as mean with the ± standard error (±SE). Figure 4C is compared using Two‐way ANOVA with Tukey. Figures 5B and 7E were analyzed using AUC followed by a Welch's T‐Test. Figure 8D was analyzed using one‐way ANOVA with Tukey. For all analyses the significance was set at ^*^ *p* < 0.05, ^**^ *p* < 0.01, ^***^ *p* < 0.001, ^****^ *p* ≤ 0.0001.

## Conflict of Interest

The authors declare no conflict of interest.

## Author Contributions

D.H. devised and performed all experiments, designed the stent, and drafted the manuscript; J.R.M., S.L.N., and S.M. conceived and directed the research; A.T. and J.R.K.M. designed and fabricated the sensors; E.R. designed the stent; T.K. aided with in vitro experiments; M.H., J.C., and N.M. conceived, directed, and fabricated the telemetry device; all authors reviewed the manuscript.

## Data Availability

The data that support the findings of this study are available on request from the corresponding author. The data are not publicly available due to privacy or ethical restrictions.
